# Time-Varying Effect of Hybrid Immunity on the Risk of Breakthrough Infection after Booster Dose of mRNA COVID-19 Vaccine: The MOSAICO Study

**DOI:** 10.3390/vaccines10081353

**Published:** 2022-08-19

**Authors:** Pietro Ferrara, Domenico Ponticelli, Roberto Magliuolo, Mario Borrelli, Beniamino Schiavone, Lorenzo Giovanni Mantovani

**Affiliations:** 1 Center for Public Health Research, University of Milan-Bicocca, 20900 Monza, Italy; 2 IRCCS Istituto Auxologico Italiano, 20165 Milan, Italy; 3 Pineta Grande Hospital, 81030 Castel Volturno, Italy

**Keywords:** mRNA COVID-19 vaccine, breakthrough infections, healthcare workers, SARS-CoV-2, smoking

## Abstract

This longitudinal observational study investigated the risk of breakthrough SARS-CoV-2 infection up to 6 months after a booster dose of an mRNA COVID-19 vaccine in infection-naïve vs. previously infected healthcare workers (HCWs), and whether this difference varied over time. A Cox proportional hazard regression model with Aalen’s additive analysis was fitted to examine the association between the risk of infections and predictor variables. Overall, we observed an incidence rate of 2.5 cases per 1000 person-days (95% confidence interval [CI] 2.0–3.0), which dropped at 0.8 per 1000 person-days (95% CI 0.3–2.0) in recipients with prior SARS-CoV-2 infection. The fitted analysis indicated an adjusted hazard ratio of 0.32 (95% CI 0.13–0.80; *p*-value = 0.01) for those with hybrid immunity with a slope that became steeply negative roughly starting from day 90. No difference was seen according to participants’ smoking habits. Characteristics of infected HCWs were also described. Our study quantifies the time-varying effects of vaccine-induced and hybrid immunity after the booster dose (during the Omicron variant predominance in Italy) and observed that the protection waned more rapidly in infection-naïve recipients starting from the third month. The results add important evidence that can be used to inform COVID-19 vaccination strategies.

## 1. Introduction

The deployment of safe and effective vaccines against the coronavirus disease 2019 (COVID-19) and the swift immunization efforts have been key to reducing the death and disease burden from COVID-19 worldwide [1,2,3]. Indeed, a considerable body of real-world evidence is providing important insights into the immunogenicity, effectiveness and safety of the vaccination against COVID-19 [2,3]. In doing so, researchers are highlighting several determinants of vaccine response, useful to exploit vaccination campaigns in specific settings and populations.

In particular, understanding predictors and duration of COVID-19 vaccine effectiveness is crucial to inform about the need for and timing of booster doses, especially in view of emerging viral variants [1,4,5].

In Italy, the administration of the third dose started in September 2021, first targeting people at greater risk for COVID-19 consequences [6]—namely, immunocompromised and elderly (older than 80 years) individuals [7]. Subsequently, it was the turn of healthcare workers (HCWs), aiming at continuing ensuring their safety at work, and protecting the tightness of whole healthcare systems [8,9,10]. However, the introduction and diffusion of the B.1.1.529 Variant of Concern (named Omicron variant and parent lineages) in the country impacted the transmissibility of SARS-CoV-2 and the occurrence of breakthrough infections [11].

Again, growing research is emerging about the comparison between naturally acquired (protection conferred by previous SARS-CoV-2 infection), vaccine-induced and hybrid immunity over time, with evidence suggesting that the latter confers protection in reducing severe outcomes, although results differed according to the studies published so far [12,13,14].

To investigate the onset of breakthrough infections for up to six months after the booster dose, and to learn whether the protection conferred by previous SARS-CoV-2 infection plays a role on these infections and their outcome, we conducted this study to determine the incidence of confirmed SARS-CoV-2 infection in a sample of HCWs, accounting for the time that had passed since the vaccination and the breakthrough infection, as well as for the role of previous natural immunity.

## 2. Materials and Methods

### 2.1. Study Design and Population

MOSAICO (“Monitoraggio di Breakthrough infectionS dopo dose Addizionale di vaccino antI-COVID-19 a mRNA in operatori sanitari”) is a longitudinal observational study designed to investigate the safety and effectiveness of booster immunization with a COVID-19 vaccine in a sample of workers of the Pineta Grande Hospital (Castel Volturno, Caserta, Italy). All of the internal workers—HCWs and non-HCWs (the latter as all hospital workers who were not in close contact with patients)—were invited to participate in the study before and after the administration of the third vaccine dose with an mRNA vaccine available (either BNT162b2 or mRNA-1273), performed according to the instruction of the Ministry of Health of Italy, where the administration of the booster to healthcare personnel started in October 2021. Participation in the study was voluntary; vaccinees were not offered any incentive and were informed about their right to withdraw at any time without penalty.

For the current research, we included participants with a follow-up for breakthrough infection onset. At the enrollment, all subjects filled a self-administered questionnaire with their demographics and professional characteristics (sex, age, professional role, smoking habits, previous laboratory confirmed SARS-CoV-2 infection, and history of COVID-19 vaccination). Subsequently, participants were asked for the authorization for tracing possible breakthrough SARS-CoV-2 infections in the hospital electronic health records for up to 6 months after the vaccination. Any SARS-CoV-2 infection diagnosed through positive RT-PCR on samples obtained from nasopharyngeal swabs starting from seven or more days after the administration of the booster vaccination was considered vaccine breakthrough infections. The 7-day lag from booster doses aligns with the breakthrough case definition provided by previous research on COVID-19 vaccine effectiveness [8,15,16].

In the case of testing positive, participants were re-contacted upon testing negative and were administered a second questionnaire specifically designed to gather information about COVID-19 symptoms and clinical management of the disease.

MOSAICO was approved by the Ethics Review Board Campania Nord (CECN.1868.2022). All participants provided written informed consent.

### 2.2. Statistical Analysis

A descriptive analysis was carried out to describe cohort characteristics and outcomes. Continuous variables were summarized as mean and standard deviation (SD), or median and interquartile range (IQR), based on their distribution. Categorical variables were expressed as absolute and relative frequency.

A Cox proportional hazard regression model was fitted to examine the association between the risk of breakthrough SARS-CoV-2 infections and predictor variables. Time-to-event data were calculated from the date of vaccine administration (booster dose), and the analysis time was censored at the date of breakthrough infection onset, or at the end of follow-up time on 31 March 2022 (max 180 days). No loss at follow-up was recorded. A stepwise backward selection procedure was followed by setting a significance level of 0.40 as criterion for variables to exit the final multivariate Cox model. The following independent variables were studied for inclusion: sex (male = 0; female = 1), age (continuous, in years), professional role (non-HCW = 0; HCW = 1), smoking habits (never/former smoker = 0; current smoker = 1; electronic cigarette (e-cigarette) user = 2); previous SARS-CoV-2 infection (no = 0; yes = 1); autoimmune disease (no = 0; yes = 1); immunosuppressive therapy (no = 0; yes = 1); type of booster vaccine (BNT162b2 = 0; RNA-1273 = 1). Results for covariates that entered the final model were reported as adjusted hazard ratio (HR) and 95% confidence intervals (CI). As a supplement to the Cox model, those covariates that entered into the final model were also plotted in an Aalen’s additive regression analysis to study their time-varying effect on the analysis time through plots of the estimated cumulative regression coefficients [17]. Lastly, a sensitive post hoc analysis was performed by adjusting predictor variables against the number of daily SARS-CoV-2 cases in any index date (inserted as cubic spline with knots at 75,000, 125,000 and 200,000 positive daily swabs), as proxy of COVID-19 transmission in the area. Data on daily cases in the study period were sourced from the national COVID-19 integrated surveillance system and referred to the Caserta province [18], where it reports that the Pineta Grande Hospital and the vast majority hospital workers and patients live. The surveillance system includes daily numbers of RT-PCR and Rapid Antigen tests for SARS-CoV-2 infection [19]. Results are discussed in the limitations section.

Secondly, in the breakthrough infection cohort, the following models were constructed: a linear regression model investigating predictors of the time-length between testing positive and testing negative (Model 1); likelihood of developing at least one COVID-19 symptom during breakthrough infection (Model 2); and likelihood of using medications due to COVID-19 symptoms (Model 3). In all multivariable models, the relevant confounders were identified through a stepwise regression strategy (*p*-value 0.4 for exit); variables examined for inclusion are listed in Supplementary Material (Section S1). In the logistic regression models, adjusted odds ratios (ORs) and 95% CIs expressed the effect estimates. Standardized regression coefficients (*β*) were presented in the linear regression model.

All statistical tests were two-tailed and a *p*-value ≤ 0.05 was considered statistically significant. Data were analyzed with statistical software STATA v. 17 and R v. 4.2.0 [20,21].

## 3. Results

A total of 320 individuals participated in the study, being the majority female (59.1%), with a median age of 38 years and being mostly HCWs (77.5%). Complete characteristics of the study population are presented in Table 1.

**Figure 1:**
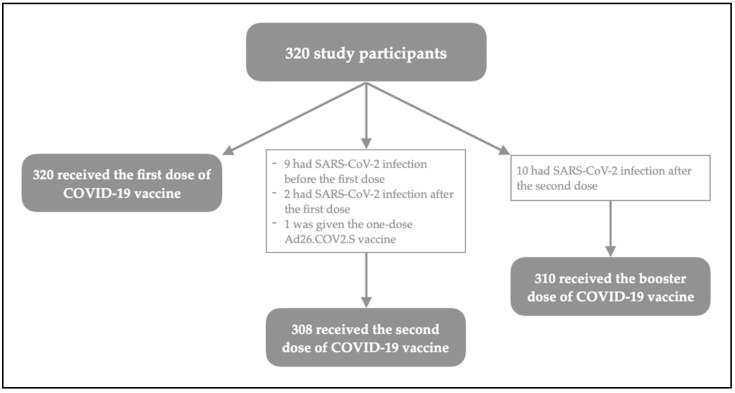
History of COVID-19 vaccination among study participants.

Overall, 60 (18.8%) subjects reported a previous infection with SARS-CoV-2, 39 of which were more than six months before the start of the vaccination campaign in Italy. Ten (3.1) of them also reported a SARS-CoV-2 positive test result after the second dose and therefore were not administered with the booster, as per the regulation of Italian Ministry of Health. Those participants were excluded from the final analyses. The flowchart of the cohort according to their vaccination status and history of previous SARS-CoV-2 infection is presented as Figure 1.

All vaccinees received an mRNA COVID-19 vaccine for boosting immunity, with 83.5% (259) who were given the BNT162b2 vaccine. Administrations took place between 2 October 2021 and 15 February 2022.

A total of 98 breakthrough infections were reported during 39,586 days of total analysis time at risk under observation, accounting for an incidence rate of 2.5 cases per 1000 person-days (95% CI 2.0–3.0). 

**Figure 2:**
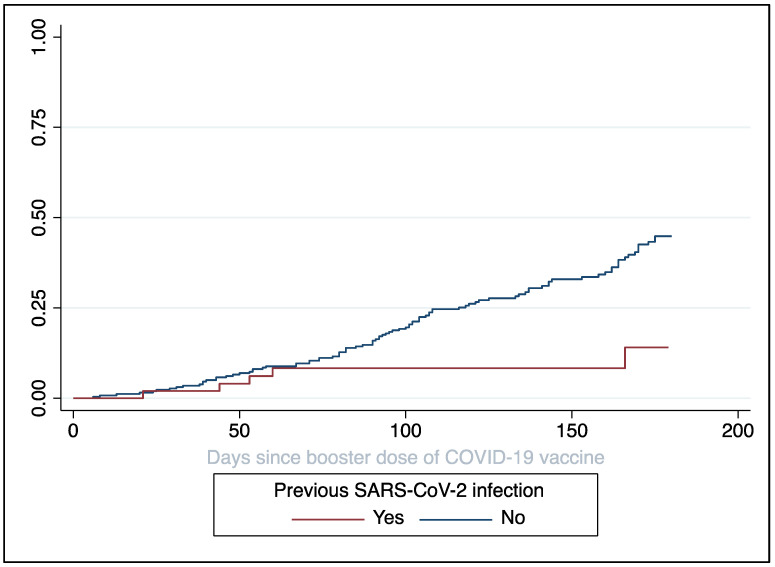
Kaplan–Meier curve for follow-up (reverse-Kaplan–Meier) according to the previous SARS-CoV-2 infection. The *y*-axis shows cumulative incidence of breakthrough infections.

Stratifying for selected characteristics, we found that the incidence dropped to 0.8 cases per 1000 person-days (95% CI 0.3–2.0) in recipients who experienced previous SARS-CoV-2 infection (irrespectively of the period), while remaining higher those who did not (rate 2.8 per 1000 person-days, 95% CI 2.3–3.4). In Figure 2, the cumulative incidence of breakthrough infections according to the previous SARS-CoV-2 positivity was described as reverse Kaplan–Meier curve.

The results of the Cox proportional hazard regression analysis indicated that the probability of breakthrough infections after the booster dose was lower in those recipients who had a prior SARS-CoV-2 infection (HR 0.32; 95% CI, 0.13–0.80; *p*-value = 0.01), were younger (HR 0.97 per year; 95% CI, 0.96–0.99; *p*-value = 0.01) and HCWs (HR 0.49; 95% CI, 0.32–0.76; *p*-value = 0.001) (Table 2).

**Figure 3:**
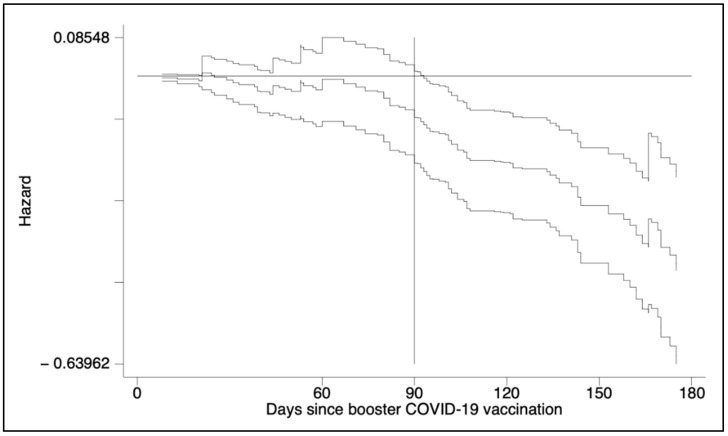
Results from Aalen’s additive regression model for censored data to the Cox regression. The plot shows how the effect of the covariate of interest (i.e., hybrid immunity) changed over time. The *y*-axis displays the baseline hazard (Intercept 0) at reference value of the covariate and the additive contributions of the time-varying difference from reference value to the hazard of breakthrough infection.

The nature of the time-varying effect of hybrid immunity is plotted in Figure 3. In detail, the Aalen’s additive analysis found that the slope of previous SARS-CoV-2 infection became negative starting from day 90 with a steep trend, while an abrupt change in slopes for professional role (HCW vs. non-HCW) and age was seen roughly at the six-month follow-up (Supplementary Figure S1).

Table 3 shows the characteristics of the 98 breakthrough infections in the study population: all were asymptomatic to mild infection and no subjects died or were hospitalized due to COVID-19 in the study population.

Table 4 shows the results for the logistic multivariate regression model’s investigating variables associated with selected characteristics of the breakthrough cases. Model 1 was designed to analyze predictors of time-length between the first SARS-CoV-2 positive swab and testing negative (that the 10 days of required quarantine in the 51.0% of cases), indicating that this increases according to the presence of an autoimmune disease (*β* coefficient = 4.85; 95% CI 1.37–8.32; *p*-value = 0.007) and decreases in females (*β* coefficient = −1.60; 95% CI −2.98–−0.22; *p*-value = 0.02). None of the collected variables was found to be associated with the likelihood of developing at least one COVID-19 symptom during breakthrough infection (Model 2), while the unique covariate predicting the likelihood of using medications (Model 3) was the presence of at least one COVID-19 symptom (OR 13.25; 95% CI, 2.69–65.30; *p*-value = 0.001).

## 4. Discussion

Real-world data from this study offer robust evidence regarding the incidence of breakthrough infections up to 6 months after a booster dose of the mRNA COVID-19 vaccine. Overall, the infection rate found in our hospital population mirrored that from similar studies, although possible rate variation across available evidence exists, mostly attributable to differences in time-to-event, settings, populations enrolled, and local vaccination policies (in terms of timing, mix-and-match of doses, etc.) [12,22,23]. The sharply increasing tendency of positive cases registered over the study period was also in line with the incidence trends of COVID-19 cases registered in the national surveillance system for Campania region during the same period [18,24].

More interestingly, this study is the first, to the best of our knowledge, that analyzed the nature of the time-varying effect of hybrid immunity—defined as two mRNA vaccine doses and previous natural infection—on the risk of breakthrough infection. The Cox proportional hazard regression analysis showed that the risk is reduced by roughly two-thirds in previous infected individuals over the 6 months following the booster immunization (during the Omicron variant predominance in Italy). Additionally, we were able to observe that this effect started roughly at the third month, allowing us to affirm that the protection against the infection waned more rapidly in infection-naïve booster recipients.

According to previous studies, people who have had SARS-CoV-2 infection showed longer-lasting benefits from a full course of vaccination. Indeed, hybrid immunity has been linked to higher levels of neutralizing antibodies and stronger adaptive immune response (mediated by B cells, T CD4+ cells, T CD8+ cells), with greater cross-protection also from SARS-CoV-2 variants [12]. However, the research available so far was mostly based on data collected on shorter follow-up periods and before the descendent lineages of the B.1.1.529 Omicron variant emerged, which have been endowed with increased infection rates [25,26,27,28]. In this sense, our findings add important evidence on the comparison between the protection conferred by either full vaccination cycle or COVID-19 vaccines plus infection, fixing the time-point for when this difference becomes crucial to protect people from COVID-19 consequences and inform future vaccination schemes.

Extending the analysis to investigate the time-varying effect of the other covariates that entered the Cox regression of MOSAICO dataset—namely, professional role, age, and smoking habits—we saw abrupt changes in the slopes for non-HCW in the fifth month of follow-up. However, this moment coincided with the loosening of the majority of community mitigation strategies in Italy enforced after the COVID-19 outbreak in 2020—including removing mandatory masks indoors, while the mandate remained for HCWs [29]. No effect of smoking (and electronic cigarette use) on risk of infection was seen in this study. Smoking’s role in SARS-CoV-2 infection, disease, and immunization has been widely studied, with findings of a robust association between a history of smoking and lower risk of infection, but higher risk of severe COVID-19 [9,30,31,32]. In our previous research designed to explore the response to the first vaccination cycle with BNT162b2 mRNA COVID-19 vaccine [2,5,8], we described a negative effect of cigarette smoking on humoral response to vaccines [33], which was further confirmed for other COVID-19 vaccines in a rapid systematic review [34]. However, since the level of vaccine-induced immunity that correlates with effective protection had not been recognized, it was difficult to learn to what extent smoking habits may affect vaccination. In this sense, the MOSAICO results detected no difference in the hazard for breakthrough infection between non-smokers and current cigarette smokers or dual users. This finding raises the question of whether smoking has an ancillary effect of partially impairing COVID-19 vaccines’ effectiveness (compared to infection risk in smokers of pre-vaccination era [31]), and fosters further research.

Describing the cohort of subjects who tested positive during the study period, most breakthrough cases were mild or asymptomatic, none required hospitalization, and the time-length between testing positive and testing negative was beyond the 10 days of required quarantine in half of the infected workers; of these, four returned to work after 3 weeks, with a significantly longer period for those with an autoimmune disease and shorter for females. Of note, the first was associated with a major risk of infection also in vaccine-naïve patients, and an impact of these conditions on vaccine response confirms the need for specific immunization strategies targeting vulnerable people [5,22,31].

A quarter of enrollees with COVID-19 decided to use antibiotics, the vast majority of which reported azithromycin use. Mass—and improper—antibiotic prescribing and use during the pandemic period is a well-studied phenomenon, particularly in Italy, where we have witnessed a high shortage of azithromycin as the Omicron-sustained incidence of SARS-CoV-2 cases increased. This is all the more alarming because a high prevalent use of antibiotics was reported in a sample of hospital workers, calling for urgent public health efforts to promote correct antimicrobials among HCWs and avoid an irredeemable *syndemic* of COVID-19 and antimicrobial resistance [35]. 

The study may be affected by limitations. First, due to its design, it did not consider all possible confounders and bias which may influence the incidence of vaccine effectiveness and the onset of breakthrough infections. However, we included those mainly associated with the outcomes, and the use of robust statistical analysis allowed us to balance factors potentially correlated to such confounders. Second, although we performed a sensitive analysis adjusting the Cox model for the number of positive daily swabs at the index date (as proxy of COVID-19 epidemiological conditions in the geographical area), results cannot be considered conclusive since there was evidence that the proportional-hazards assumption had been violated (test for proportional-hazards assumption with *p*-value < 0.0001 for both (i) global model and (ii) number of positive swabs), likely attributable to an inadequate sample size for this type of analysis. Third, the MOSAICO cohort did not allow us to investigate the role of hybrid immunity in other vulnerable populations of older persons with coexisting illnesses, being the cohort constituted mostly by young and healthy workers. Fourth, the study design cannot precisely assess the contribution of the vaccination versus natural immunity, also considering differences according to vaccination strategies, individual characteristics, severity of previous COVID-19 infections, and increased immune escape of new viral variants. Lastly, although the most reported COVID-19-like symptoms are generally associated with infection and diseases, we were not able to measure the duration and persistence of symptoms, thus we cannot completely describe the impact of hybrid immunity on COVID-19.

## 5. Conclusions

In conclusion, our research adds important real-world data to the ongoing vaccination campaigns against COVID-19, and allowed us to determine the correlate of protection against breakthrough infections conferred by hybrid immunity—intended as two vaccine doses plus infection—over a 6-month period after booster dose. The data collected confirm the need for continuous monitoring of vaccine-induced immunogenicity over longer follow-up periods in order to provide real-world data that may support vaccination policies.

## Figures and Tables

**Table 1:** Study population.

	N (%)
Total vaccinees	320
Age *	38 (32–50)
Sex	
Male	131 (40.9)
Female	189 (59.1)
Role	
HCWs	248 (77.5)
Non-HCWs	72 (22.5)
Cigarette smoker	
Never	178 (55.6)
Current	121 (37.8)
Former	21 (6.6)
E-cigarette user	50 (15.6)
Of which, dual users	49 (15.3)
Health status	
Previous SARS-CoV-2 infection	60 (18.8)
Autoimmune disease	10 (3.1)
Immunosuppressive therapy	5 (1.6)

* Summarized by median and interquartile range (IQR). Abbreviations: HCWs, healthcare workers; SARS-CoV-2, Severe Acute Respiratory Syndrome Coronavirus 2.

**Table 2:** Cox multivariate regression model indicating associations between the occurrence of breakthrough infection with SARS-CoV-2 and characteristics evaluated (N = 309).

Variable	Hazard Ratio	SE	95% CI	*p*-Value
*Log likelihood = −507.17; χ2 = 27.39 (4 df); p-value < 0.0001*				
Previous infection with SARS-CoV-2				0.01
No	*Ref.*	-	-	
Yes	0.32	0.15	0.13–0.80	
Age (continuous, in years)	0.97	0.01	0.96–0.99	0.01
Professional role				0.001
Non-HCWs	*Ref.*	-	-	
HCWs	0.49	0.11	0.32–0.76	
Smoking habits				0.08
Never/former smoker	*Ref.*	-	-	
Cigarette smoker	0.61	0.17	0.35–1.06	
E-cigarette user/Dual user	*Omitted for p-value > 0.4*			

Abbreviations: HCWs, healthcare workers; SARS-CoV-2, Severe Acute Respiratory Syndrome Coronavirus 2; *Ref*., reference category; 95% CI, 95% confidence interval; SE, standard error; df, degrees of freedom.

**Table 3:** Characteristics of the breakthrough infection in the study population (N = 98).

	N (%)
Contact with confirmed COVID-19 case	57 (57.6)
Time length from testing positive to negative (in days) *	11.0 ± 3.6
COVID-19 symptoms	
At least one	79 (80.6)
Nasal congestion or runny nose	35 (35.7)
Muscle/joint or body pains	34 (34.7)
Fever/chills	33 (33.7)
Cough	31 (31.6)
Sore throat	25 (25.5)
Headache	20 (20.4)
Shortness of breath or difficulty in breathing	10 (10.2)
Asthenia/fatigue/weakness	8 (8.2)
Ageusia (loss of sense of taste)	6 (6.1)
Anosmia (loss of smell)	6 (6.1)
Tachyarrhythmia	2 (2.0)
Vertigo	2 (2.0)
Neuralgia	1 (1.0)
Use of medication ^§^	
At least one	51 (53.1)
Paracetamol	22 (22.9)
Non-steroidal anti-inflammatory drug	9 (9.4)
Prednisone	14 (14.6)
Betamethasone	2 (2.1)
Dexamethasone	1 (1.0)
Unknown glucocorticosteroid	4 (4.2)
Azithromycin	15 (15.6)
Amoxicillin/clavulanic acid	3 (3.1)
Clarithromycin	1 (1.0)
Unknown antibiotic	5 (5.2)
Bromhexine	1 (1.0)
Oxymetazoline	1 (1.0)
C and/or D vitamin supplement	21 (21.9)
Lactoferrin	2 (2.1)

* Summarized by mean ± standard deviation (SD). *^§^* Percentage was calculated on 96 observations due to missing data.

**Table 4:** Multivariate regression models predicting the characteristics of the breakthrough infections.

Model 1: Time-length between testing positive and negative (N = 98)				
**Variable**	**Coefficient**	**SE**	**95% CI**	***p*-value**
*F* *(3,94) = 6.15; p-value = 0.0007; R^2^ = 0.16; adjusted R^2^ = 0.14*				
Autoimmune disease				0.007
No	*Ref.*	-	-	
Yes	4.85	1.75	1.37–8.32	
Sex				0.02
Male	*Ref.*	-	-	
Female	−1.60	0.70	−2.98–−0.22	
Smoking habits				0.13
Never/former smoker	*Ref.*	-	-	
Cigarette smoker	*Omitted for p-value > 0.4*	-	-	
E-cigarette user/Dual user	1.38	0.17	−0.41–3.17	
Model 2: Likelihood of develop at least a COVID-19 symptom during breakthrough infection (N = 89)				
**Variable**	**Odds Ratio**	**SE**	**95% CI**	***p*-value**
*Log likelihood = −42.51; χ2 = 7.27 (3 df); p-value = 0.06*				
*Age* (continuous, in years)	1.05	0.3	1.00–1.11	0.05
Time-length between testing positive and negative (continuous, in days)	1.11	0.10	0.94–1.32	0.23
Smoking habits				0.29
Never/former smoker	*Ref.*	-	-	
Cigarette smoker	*Omitted for p-value > 0.4*	-	-	
E-cigarette user/Dual user	0.46	0.33	0.11–1.88	
Model 3: Likelihood of using medications due to COVID-19 symptoms (N = 91)				
**Variable**	**Odds Ratio**	**SE**	**95% CI**	***p*-value**
*Log likelihood = −53.23; χ2 = 19.67 (4 df); p-value = 0.0006*				
Presence of at least one COVID-19 symptom				0.001
No	*Ref.*	-	-	
Yes	13.25	10.78	2.69–65.30	
Sex				0.08
Male	*Ref.*	-	-	
Female	2.38	1.18	0.91–6.27	
Smoking habits				0.31
Never/former smoker	*Ref.*	-	-	
Cigarette smoker	1.92	1.24	0.54–6.81	
E-cigarette user/Dual user	*Omitted for p-value > 0.4*	-	-	

Abbreviations: *Ref*., reference category; 95% CI, 95% confidence interval; SE, standard error; df, degrees of freedom.

## Data Availability

Data and supporting materials associated with this study will be provided upon request by contacting the corresponding author.

## Supplementary Materials

The following are available online at https://www.mdpi.com/article/10.3390/vaccines10081353/s1, Section S1. Appendix to statistical analysis; Section S2. Appendix to Results. Figure S1: Results from Aalen’s additive regression model for censored data to the Cox regression of MO-SAICO dataset. The plots show how the effect of the covariates that entered the final model changed over time. The *y*-axis displays the baseline hazard (Intercept 0) at reference values of covariates and the additive contributions of the time-varying differences from reference values to the hazard of breakthrough infection.
