# Supplementary material for: Time-Varying Effect of Hybrid Immunity on the Risk of Breakthrough Infection after Booster Dose of mRNA COVID-19 Vaccine: The MOSAICO Study
Source: Vaccines (Basel). 2022 Aug 19;10(8):1353. doi: 10.3390/vaccines10081353 (PMC9413553; doi:10.3390/vaccines10081353)
Supplement: Supplementary file 1 [file vaccines-10-01353-s001.zip › vaccines-1851797-supplementary.pdf]

vaccines

MDPI

Supplementary material

Section S.1. Appendix to statistical analysis

In Model 1: Time-length between positivity and negativization (Table 4) the following independent variables were examined for inclusion: sex (male = 0; female = 1), age (continuous, in years), smoking habits (never/former smoker = 0; current smoker = 1; e-cigarette user = 2); previous SARS-CoV-2 infection (no = 0; yes = 1); autoimmune disease (no = 0; yes = 1); immunosuppressive therapy (no = 0; yes = 1); and type of booster vaccine (BNT162b2 = 0; RNA-1273 = 1).

In Model 2: Likelihood of develop at least a COVID-19 symptom during breakthrough infection (Table 4) the following independent variables were studied for inclusion: sex (male = 0; female = 1), age (continuous, in years), smoking habits (never/former smoker = 0; current smoker = 1; e-cigarette user = 2); previous SARS-CoV-2 infection (no = 0; yes = 1); autoimmune disease (no = 0; yes = 1); immunosuppressive therapy (no = 0; yes = 1); type of booster vaccine (BNT162b2 = 0; RNA-1273 = 1); and time-length between positivity and negativization (continuous, in days).

In Model 3: Likelihood of using medications due to COVID-19 symptoms (Table 4) the following independent variables were studied for inclusion: sex (male = 0; female = 1), age (continuous, in years), smoking habits (never/former smoker = 0; current smoker = 1; e-cigarette user = 2); previous SARS-CoV-2 infection (no = 0; yes = 1); autoimmune disease (no = 0; yes = 1); immunosuppressive therapy (no = 0; yes = 1); time-length between positivity and negativization (continuous, in days); presence of at least one COVID-19 symptom (no = 0; yes = 1), and professional role (nonHCW = 0; HCW = 1).

Section S.2. Appendix to Results

Vaccines **2022**, *10*, 1353. https://doi.org/10.3390/vaccines10081353 www.mdpi.com/journal/vaccines

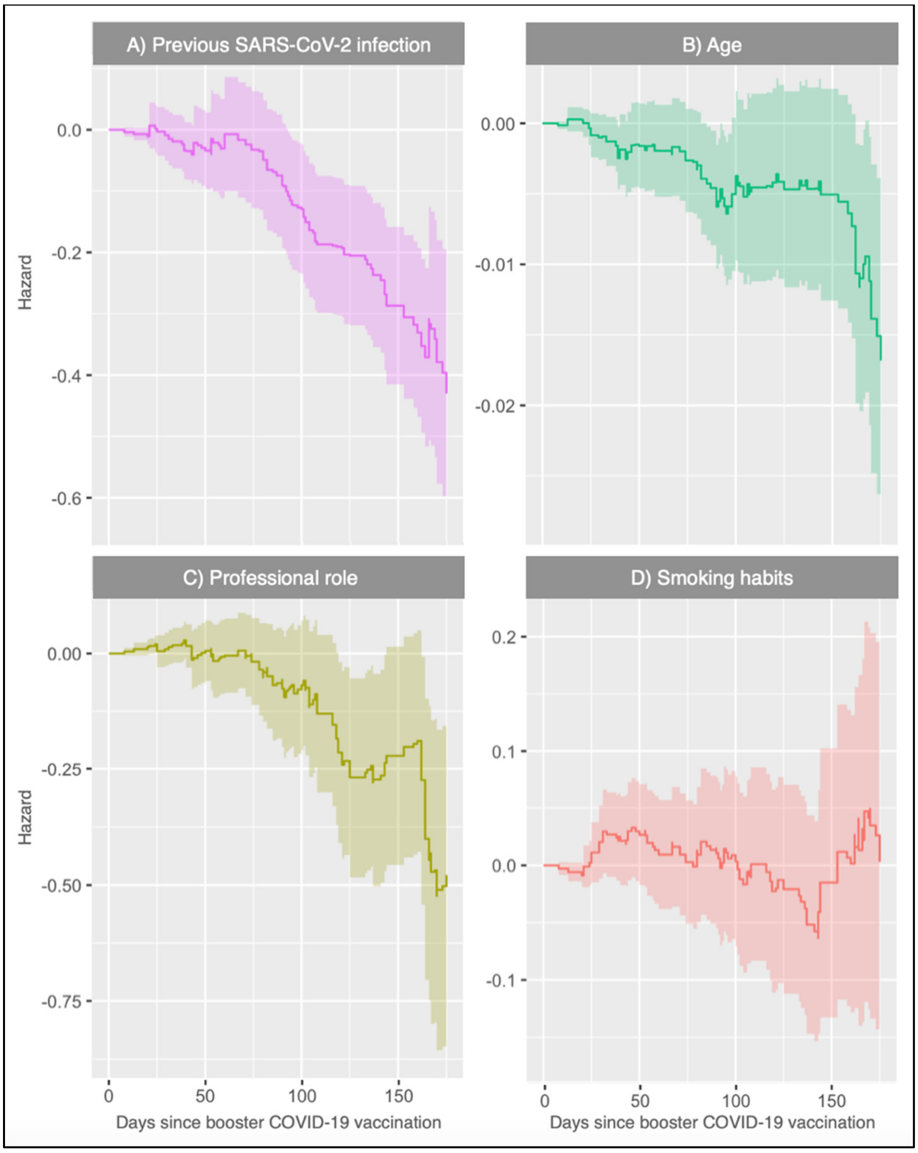

Figure S1. Results from Aalen's additive regression model for censored data to the Cox regression of MOSAICO dataset. The plots show how the effect of the covariates that entered the final model changed over time. The y-axis displays the baseline hazard (Intercept 0) at reference values of covariates and the additive contributions of the time-varying differences from reference values to the hazard of breakthrough infection.
